# Antifriction and Antiwear Effect of Lamellar ZrS2 Nanobelts as Lubricant Additives

**DOI:** 10.3390/nano9030329

**Published:** 2019-03-01

**Authors:** Wei Tang, Chuang Yu, Shaogang Zhang, Songyong Liu, Xingcai Wu, Hua Zhu

**Affiliations:** 1 School of Mechatronic Engineering, China University of Mining and Technology, Xuzhou 221116, China; chensicumt@126.com (S.Z.); liusongyong@163.com (S.L.); zhuhuacumt@126.com (H.Z.); 2 School of Chemistry and Chemical Engineering, Nanjing University, Nanjing 210023, China; wuxingca@nju.edu.cn

**Keywords:** lamellar, ZrS_2_, lubricant additives, antifriction, antiwear

## Abstract

In this study, the tribological behavior of lamellar ZrS_2_ nanobelts as lubricant additives was investigated under different concentrations, normal load, velocity, and temperature. The friction and wear tests were performed using a tribometer and with a reciprocating motion. The results indicate that the lamellar ZrS_2_ nanobelt additives can effectively reduce the coefficient of friction and running-in time during the running-in period. With the addition of ZrS_2_, the wear volumes decrease significantly. The wear is mostly influenced by the tribological performance throughout the running-in period. The lower the running-in time and coefficient of friction are during the running-in period, the less amount of wear is shown. ZrS_2_ can significantly increase the load-carrying capacity of oil. The 1.0 wt% concentration of ZrS_2_ yields the best antifriction effect, antiwear performance, and load-carrying capacity. The ZrS_2_ additives can increase the working temperature of the oil. The friction-reducing and antiwear mechanisms of lamellar ZrS_2_ were discussed.

## 1. Introduction

Two-dimensional (2D) lamellar structured materials have strong covalent intralayer bonding but weak van der Waals interlayer bonding. This distinctive layered structure can significantly improve the tribological properties of materials and has considerable applications in solid lubrication, lubrication additives, and self-lubricating polymer materials [1,2,3,4].

The transition metal dichalcogenides MX_2_ (M = Mo, W, Nb, Zr, V, Ta, Ti, and Hf; X = S and Se) have similar 2D layered structures to graphene, which has increased research interest, and these metals have been widely studied as lubricant additives in oil [5,6,7,8]. Many studies have shown that due to the distinctive layered structure, transition metal disulfide has considerable friction reduction and antiwear performance. Jamison and Cosgrove [9] investigated the disulfides of the second and third row transition metals for their ability to function as solid lubricants. Their studies showed the lubricating performance of the layered transition metal disulfides was governed by the axial ratio of the lattice parameters. The study of Kogovšek et al. [10] indicated that the addition of MoS_2_ nanotubes to the base oil can significantly improve the friction behavior under the boundary and mixed lubrication conditions. From the investigation of Aldana et al. [11], it was found that, with the addition of WS_2_ nanoparticles to the base oil, both wear and friction coefficient can be reduced by around 70%. Zhang et al. [12] produced various 2D nanosheets (h-BN, MoS_2_, MoSe_2_, WS_2_, and graphene) as aqueous dispersions. It was found that these aqueous lubricants can achieve a friction coefficient as low as 0.02 and even achieve superlubricity under certain working conditions. 

Among dichalcogenides, ZrS_2_ has the same 2D layered structures as the above materials [9,13]. It is hypothesized that the lamellar ZrS_2_ can reduce friction and wear when used as a lubricant additive. The layered ZrS_2_ has been shown to have significant applications in semiconductor nanodevices [14,15,16]. However, in this previously reported study, the lamellar ZrS_2_ is rarely studied as a lubricant additive. In this study, the tribological behaviors of lamellar ZrS_2_ a as lubricant additive were investigated under different concentration, normal load, velocity, and temperature. The friction and wear tests were carried out using a tribometer. Oleic acid was used as a surfactant to improve the dispersing properties of the lamellar ZrS_2_. The coefficient of friction, wear volume, and maximum nonseizure load were discussed under different lubricated conditions. The related antiwear and antifriction mechanisms were also discussed. The objective of this study is to understand the antifriction and antiwear effect and the related lubrication mechanisms of the ZrS_2_. It is expected that this research can proved an alternative 2D additive material in lubrication.

## 2. Experimental Details

### 2.1. Lubrication Preparation

**Figure 1:**
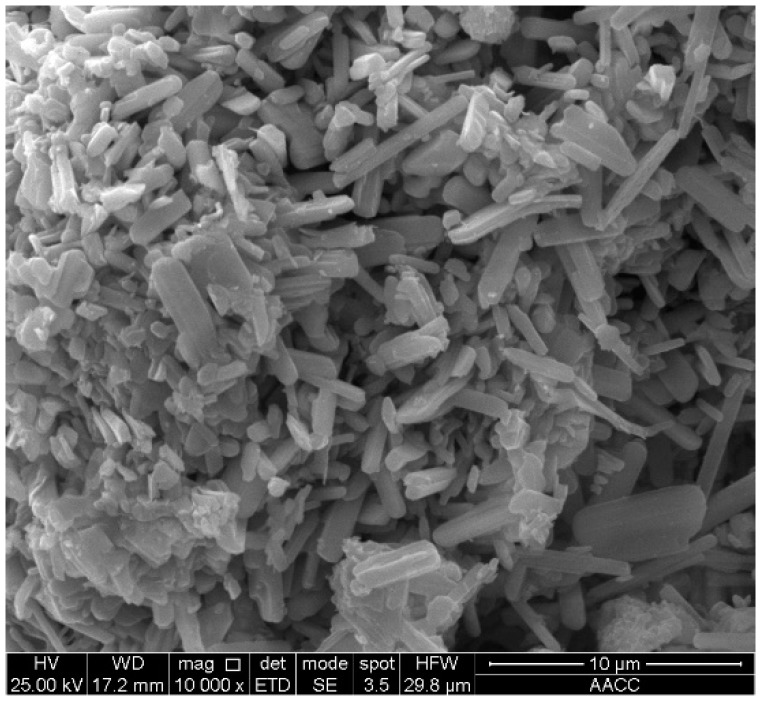
Scanning Electron Microscope (SEM) images of lamellar ZrS_2_ nanobelts.

**Figure 2:**
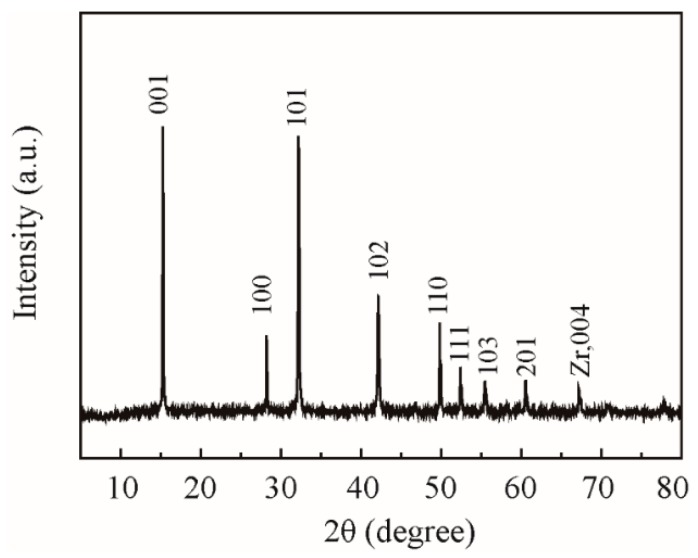
XRD pattern of lamellar ZrS_2_ nanobelts.

The 2D lamellar ZrS_2_ nanobelts (99.90%) were synthesized with a combined process of chemical vapor transport and vacuum pyrolysis, the detailed methods of which are shown in reference [5]. Figure 1 shows the images of ZrS_2_. Figure 2 shows the X-ray diffraction (XRD) pattern of ZrS_2_, which confirms the characteristic diffraction peaks.

**Figure 3:**
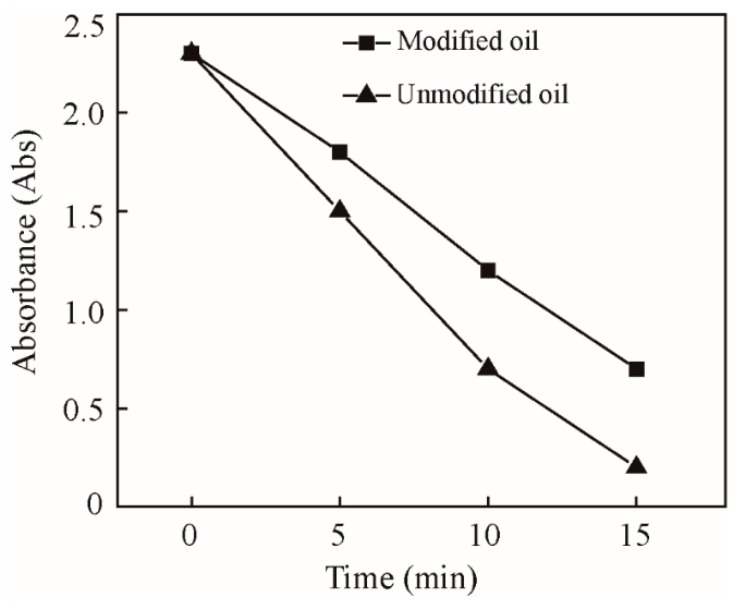
Absorbency curve of the oil with time.

Oleic acid works as a surfactant of ZrS_2_ in order to improve the dispersing properties [17]. The ZrS_2_ and 1.0 wt% oleic acid were mixed with the base oil (liquid paraffin, supplied by Sigma-Aldrich, (Shanghai, China) to get the different lubricant concentrations. The mixture was stirred to make a uniform suspension using a megnetic stirrer at 1500 rpm for 60 min at 60 °C. The suspension was then processed by ultrasonication with stirring for 30 min to breakdown any remaining agglomeration, leading to the desired samples with different contents of ZrS_2_. In order to evaluate the dispersibility of the suspension, the dispersive solution was put into the centrifugal tube for centrifugal test. After centrifugation for a certain time, the upper lubricant of the centrifugal tube was taken for absorbance test. Figure 3 shows the dispersibility of oil evaluated by the optical absorbance spectrum with time. It shows that oil dispersed with modified ZrS_2_ is more stable than the oil with unmodified ZrS_2_. The average size of ZrS_2_ in the oil is 2–5 µm in length, 0.5–2 µm in width, and 50–200 nm in thickness.

### 2.2. Tribological Tests

**Figure 4:**
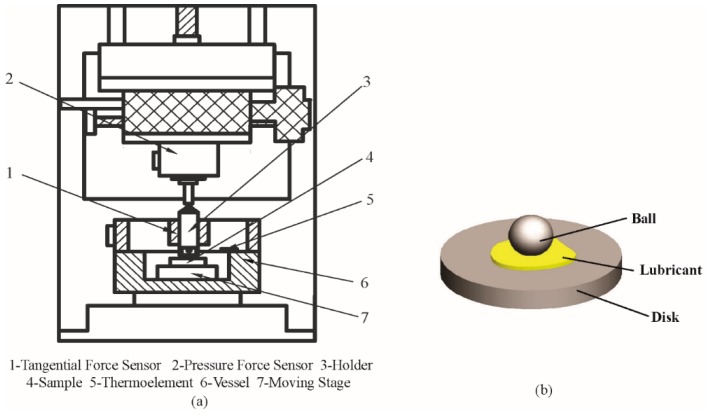
Schematic of (**a**) testing system and (**b**) ball-on-disk configuration.

The tribological tests were carried out using a tribometer (UMT-2, Center for Tribology Inc., Campbell, CA, USA) with a ball-on-disk configuration. The schematic of the testing system is shown in Figure 4. Table 1 shows the parameters of the ball-on-disk configurations. Table 2 shows the material chemical compositions of the ball-on-disk configurations. The reciprocating friction and wear tests were carried out under different lubricated conditions. The average test values were obtained based on the four-times-repeated test. 

During the test, a 0.2 mL oil sample was added to the sample surface every 30 min to ensure proper lubrication. After the test, all samples were cleaned and dried to measure the width and depth of the wear scar using a surface roughness tester. The final reported wear volume for the samples was the average of five points on the wear scar. 

The extreme pressure properties of oil with different concentrations of ZrS_2_ were evaluated using a four-ball tester (MRS-10P, Jinan Testing Machine Factory, Jinan, China) according to Chinese national standard GB/T 12583-90.

### 2.3. Characterization

The morphology of the wear scars and their surface roughness characteristics were analyzed with a high-powered microscope (YSDS, Beijing, China) and a surface roughness tester (Taimig, Shanghai, China). Elemental analysis on the worn surfaces was analyzed using energy dispersive X-ray spectroscopy (EDS, FEI, Hillsboro, OR, USA).

## 3. Results and Discussion

### 3.1. Influence of the Concentrations of Disulfide Zirconium

According to the literature regarding the tribological behavior of layered nanoparticles as lubricant additive [18,19,20], most concentration that shows good antifriction and antiwear is around 0.1 wt%–0.5 wt%. Finally, four concentrations of ZrS_2_ in base oil (0.1 wt%, 0.5 wt%, 1.0 wt%, and 1.5 wt%) were chosen.

The friction and wear tests were carried out with different concentrations of ZrS_2_ (0 wt%, 0.1 wt%, 0.5 wt%, 1.0 wt%, and 1.5 wt%) under a normal load of 20 N (average contact pressure of 2.45 GPa), a velocity of 40 mm/s, and normal ambient conditions (25 °C, relative humidity (RH) 50%–60%). The friction and wear tests were carried out for 0.5 h and 8 h, respectively.

#### 3.1.1. Friction Reducing and Antiwear Effect

**Figure 5:**
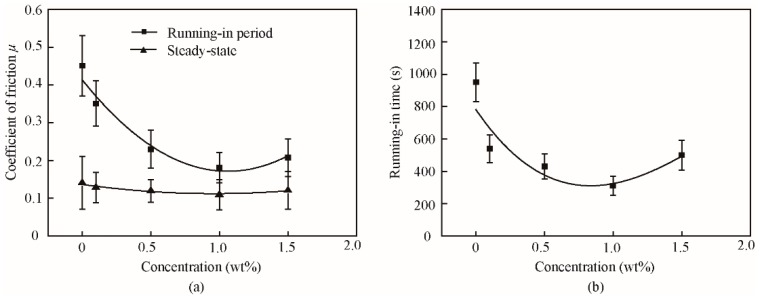
(**a**) friction coefficient and (**b**) running-in time of the base oil and that with different concentrations of lamellar ZrS_2_ nanobelt additives

**Figure 6:**
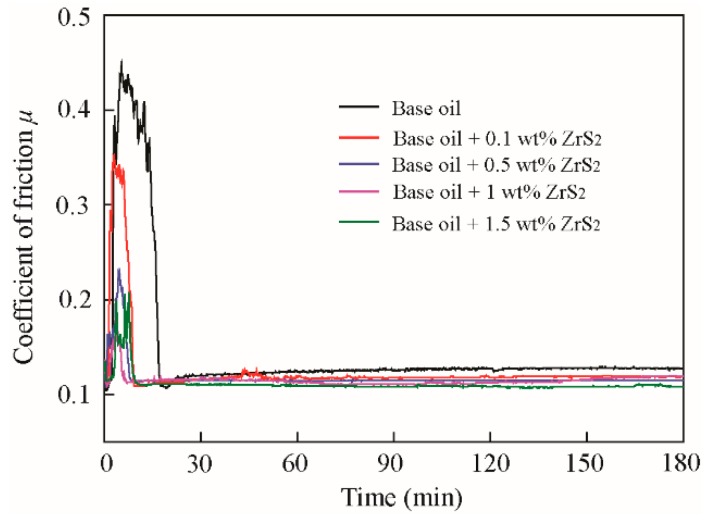
Typical friction coefficient of the base oil and that with different concentrations of lamellar ZrS_2_ nanobelt additives.

The average and typical friction coefficients of the base oil and that with different concentrations of lamellar ZrS_2_ nanobelt additives, are shown in Figure 5 and Figure 6.

The results show that for all oil samples, there are running-in periods and a steady-state. During the running-in period, the friction coefficient shows a large value and obvious vibration. After the running-in period, the coefficient of friction is reduced to a low and stable value, where the friction transitions to the steady-state. 

With the addition of 0.1 wt%, 0.5 wt%, 1.0 wt%, and 1.5 wt% ZrS_2_ nanobelts, the coefficient of friction in the running-in period decreases by approximately 22%, 49%, 60%, and 54%; additionally, the running-in time decreases by approximately 43%, 55%, 67%, and 47%, respectively, when compared to the base oil. This result indicates that the addition of ZrS_2_ can effectively decrease the friction coefficient in the running-in period and reduce the running-in time. 

In the steady-state, with the addition of 0.1 wt%, 0.5 wt%, 1.0 wt%, and 1.5 wt% ZrS_2_ nanobelts, the coefficient of friction decreases by approximately 9%, 15%, 22%, and 14%, respectively. The friction reducing effect of ZrS_2_ in the steady-state is less obvious than that in the running-in period. The 1.0 wt% concentration of ZrS_2_ yields the best antifriction performance.

**Figure 7:**
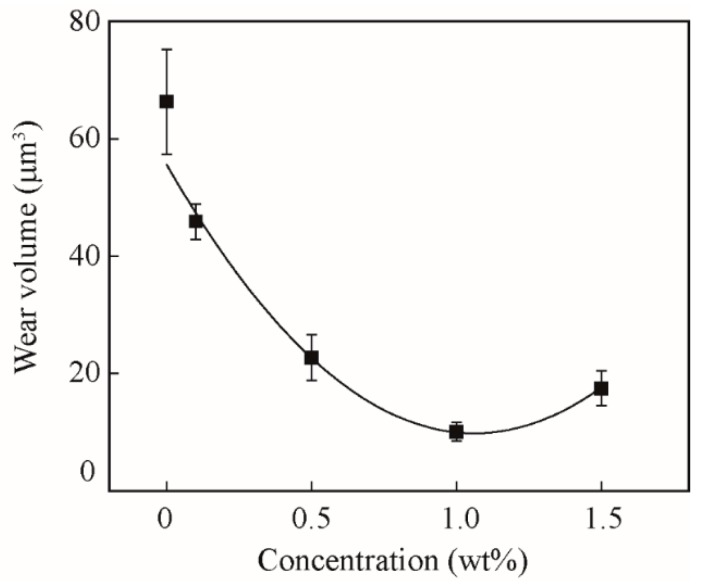
Wear volume of surfaces lubricated by oils with different concentrations of lamellar ZrS_2_ nanobelt additives.

**Figure 8:**
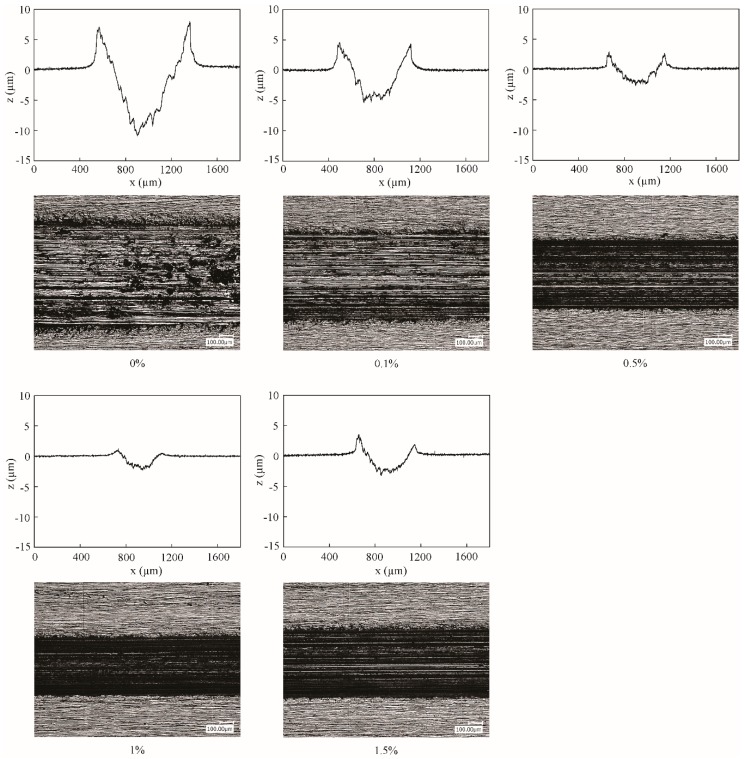
Two dimension (2D) profile curves and images of the wear scars lubricated by the base oil and that with different concentrations of lamellar ZrS_2_ nanobelt additives. *x* is scan length, *z* is depth.

Figure 7 shows the wear volume of surfaces lubricated by oils with different concentrations of lamellar ZrS_2_ nanobelt additives. The surface images and section profiles of the wear scar are shown in Figure 8.

The results show that, with the addition of ZrS_2_, the wear volumes decrease significantly. The surfaces lubricated by the base oil show the largest wear volume of 66 µm^3^. The surfaces lubricated by the oil with 1.0 wt% ZrS_2_ show the smallest wear volume of 10 µm^3^, which decrease by 85%, when compared with the base oil. The results suggest that the addition of ZrS_2_ can considerably decrease the wear. The 1.0 wt% ZrS_2_ shows the optimal antiwear effect. Since the friction reducing effect is less obvious for ZrS_2_ in the steady-state, the wear is mostly influenced by the tribological performance in the running-in period. The lower the running-in time and coefficient of friction are in the running-in period, the less the amount of wear is shown.

**Figure 9:**
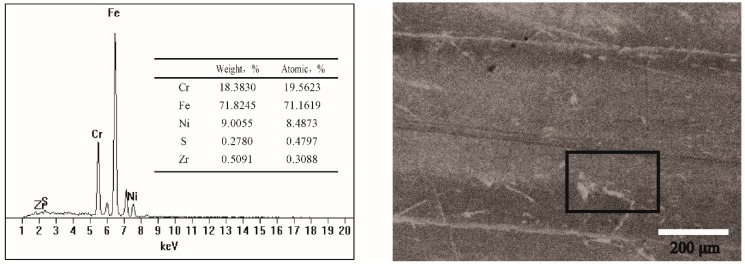
EDS of the wear scar lubricated by the base oil with lamellar ZrS_2_ nanobelt additives.

The EDS data of the wear scars lubricated by the base oil and the different concentrations of ZrS_2_ additives are shown in Figure 9. The results show that all surface lubricated by the oil with ZrS_2_ additive has amounts of Zr element.

**Figure 10:**
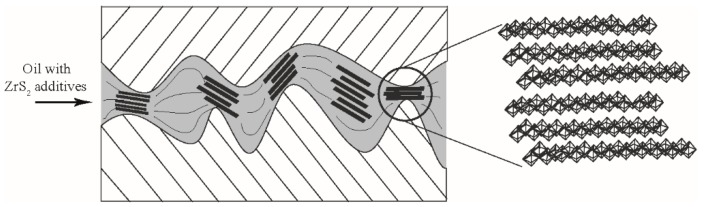
Schematic diagram of friction-reducing of lamellar ZrS_2_ nanobelts additive.

According to the references [3,4,8,21], the friction and wear reduction effects of 2D nanomaterials as lubricant additive are mainly due to the interlayer sliding, filling of the furrows, and formation of surface film. It is speculated that the small size allows ZrS_2_ to easily enter into the sliding contact area. Due to the weak van der Waals interlayer bonding, the layered structure of ZrS_2_ can easily produce interlayer sliding, resulting in the desired antifriction performance, as shown in Figure 10. ZrS_2_ nanobelts also can smooth the surfaces by filling the furrows on the worn surface. This outcome can help to reduce the contact pressure and plastic deformation, which improves the antiwear effect. 

#### 3.1.2. Extreme Pressure Properties

**Figure 11:**
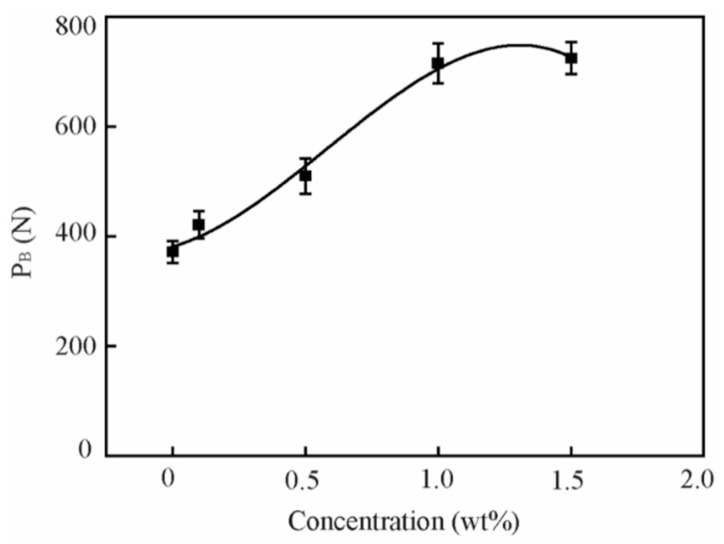
Maximum nonseizure load of the base oil and that with different concentrations of ZrS_2_ additives.

Figure 11 shows the maximum nonseizure load (PB) of the base oil and that with different concentrations of the ZrS_2_ additive. 

With the addition of 0.1 wt%, 0.5 wt%, 1.0 wt%, and 1.5 wt% ZrS_2_ nanobelts, PB values increase by approximately 13%, 37%, 92%, and 95%, respectively, when compared with the base oil. The PB value of the lubricant increases remarkably when the ZrS_2_ concentration increases from 0 to 1.0 wt%. When the concentration increases further, the PB value increases slightly. The results suggest that ZrS_2_ can significantly increase the load-carrying capacity of oil and that 1.0 wt% is the best concentration that improves the load-carrying capacity of oil.

### 3.2. Influence of Temperature

The test was carried out with 1.0 wt% ZrS_2_ with a velocity of 40 mm/s, a normal load of 20 N, and different temperatures (25 °C, 50 °C, 100 °C, 150 °C, 200 °C and 250 °C). 

**Figure 12:**
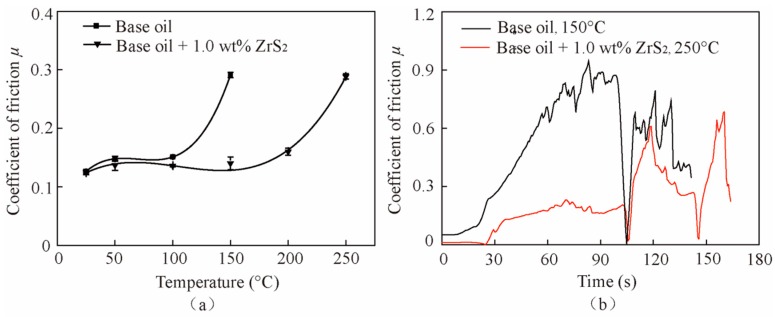
Friction coefficient of the base oil and that with 1.0 wt% ZrS_2_ additives with different temperatures: (**a**) average friction coefficient and (**b**) typical friction coefficient curves above the critical temperature.

Figure 12a shows the friction coefficient of the base oil and that with the 1.0 wt% ZrS_2_ additive at different temperatures. The results show that the critical temperatures are 100 °C and 200 °C for the base oil and for the base oil with 1.0 wt% ZrS_2_ nanobelt additives, respectively, indicating that the ZrS_2_ additives increase the working temperature of the oil. Below the critical temperature, the friction coefficient of oil with 1.0 wt% ZrS_2_ additives decreases by 1.7%, 7.4% and 10% at the temperatures of 25 °C, 50 °C and 100 °C, respectively, compared with that of the base oil. Above the critical temperature, the friction coefficient increases sharply and clearly fluctuates, as shown in Figure 12b.

**Figure 13:**
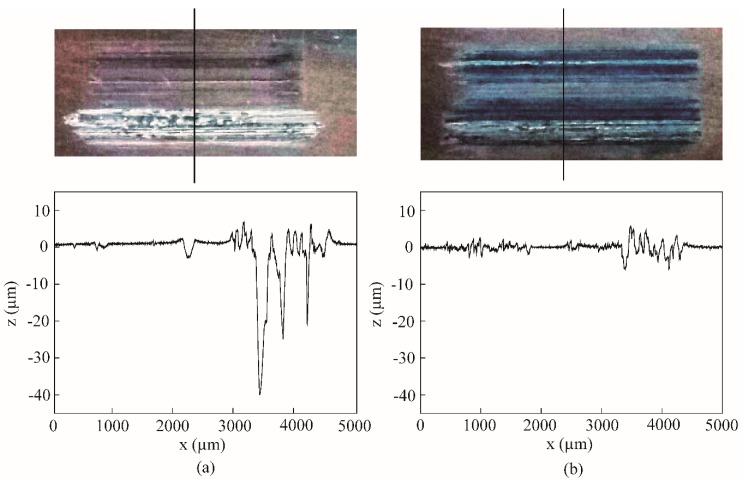
Wear scar images and 2D profile curves of worn surface lubricated by (**a**) the base oil and (**b**) the base oil with 1.0 wt% ZrS_2_ additives at 150 °C.

Figure 13 shows the wear scar images and 2D profile curves of the worn surface lubricated by the base oil and the oil with 1.0 wt% ZrS_2_ at 150 °C. In Figure 13a, it can be seen that there are deep furrows on the sample surfaces, indicating that the oil lost its good lubrication effect at 150 °C. Due to the viscosity reduction and oil evaporation with the increase in temperature, the intermolecular forces in the oil are weakened, and the lubricating film cannot be formed during sliding; thus, friction and wear greatly increase as the temperature increases. However, at higher temperatures, the layered ZrS_2_ additives can still be attached to the contact surfaces and function as lubrication.

### 3.3. Influence of Normal Load and Velocity

The influence of normal loading was tested under a velocity of 40 mm/s with different normal loads (10 N, 15 N, 20 N, 25 N, and 30 N). The influence of velocity was tested under a normal load of 20 N and different velocities (10 mm/s, 20 mm/s, 40 mm/s, 60 mm/s, and 80 mm/s). The tests were carried out under normal ambient conditions (25 °C, RH 50–60%).

**Figure 14:**
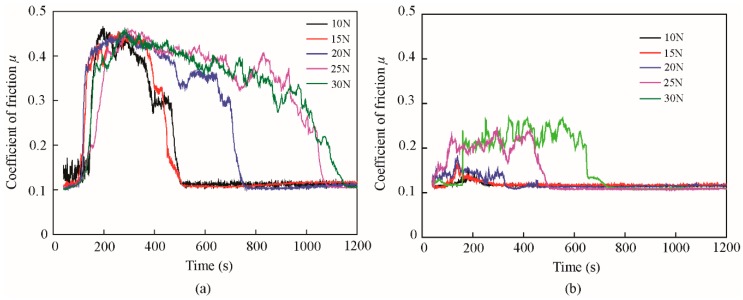
Typical friction coefficient of (**a**) the base oil and (**b**) that with 1.0 wt% ZrS_2_ nanobelt additives with different normal loads.

**Figure 15:**
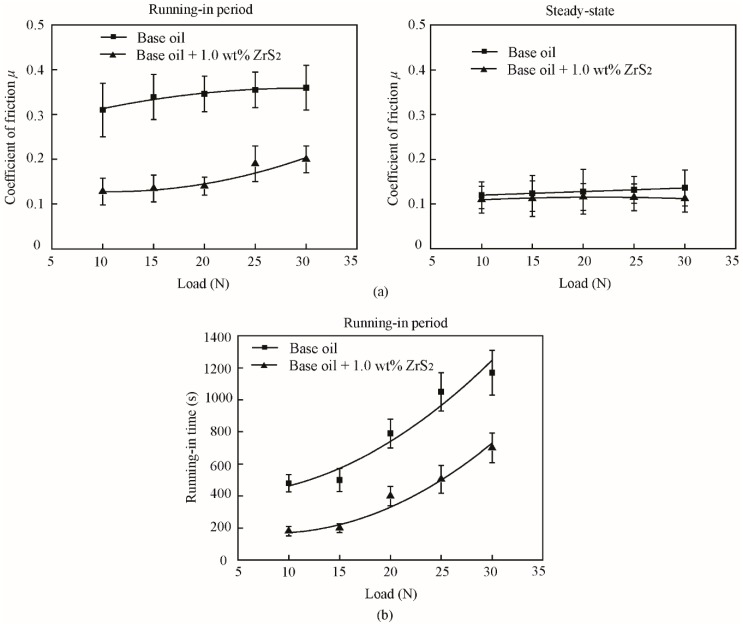
(**a**) Average friction coefficient and (**b**) running-in time of the base oil and that with 1.0 wt% ZrS_2_ nanobelt additives under different normal loads.

The typical and average friction coefficients of the base oil and that with 1.0 wt% ZrS_2_ additives under different normal loads are shown in Figure 14 and Figure 15.

The results shows that in the running-in period, with the addition of 1.0 wt% ZrS_2_, the coefficient of friction decreases by approximately 59%, 60%, 60%, 46% and 44%, and the running-in time decreases by approximately 63%, 60%, 65%, 52% and 40% under applied loads of 10 N, 15 N, 20 N, 25 N and 30 N, respectively, compared with those of the base oil. In the steady-state, the addition of ZrS_2_ can reduce the coefficient of friction under all normal loading conditions, but the friction reducing effect is not obvious compared with the effect in the running-in period. During the running-in period, the friction coefficient and running-in time of all oil samples increased with the increase in the normal load. In the steady-state, the friction coefficient of the base oil slightly increased with the increase in the normal load and oil with the addition of 1.0 wt% ZrS_2_, which was kept nearly constant.

**Figure 16:**
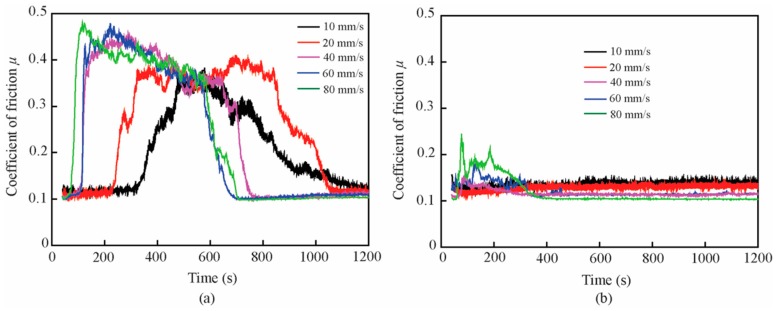
Typical friction coefficient of (**a**) base oil and (**b**) with 1.0 wt% ZrS_2_ nanobelt additives under different velocities

**Figure 17:**
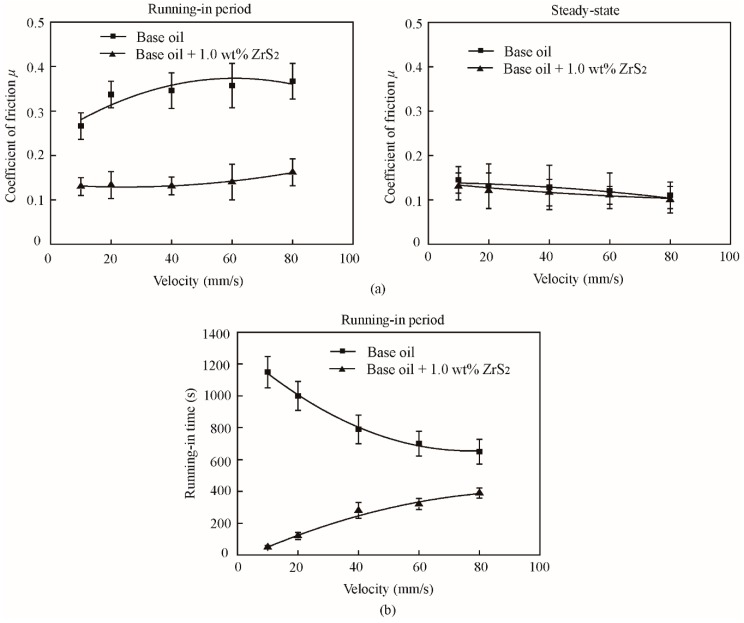
(**a**) Average friction coefficient and (**b**) running-in time of the base oil and that with 1.0 wt% ZrS_2_ nanobelt additives under different velocities.

The typical and average friction coefficients of the base oil and that with the 1.0 wt% ZrS_2_ additive at different velocities are shown in Figure 16 and Figure 17.

The results show that, in the running-in period and with the addition of 1.0 wt% ZrS_2_, the coefficient of friction decreases by approximately 51%, 61%, 62%, 61% and 56% and the running-in time decreases by approximately 96%, 88%, 65%, 54% and 40% at the velocities of 10 mm/s, 20 mm/s, 40 mm/s, 60 mm/s, and 80 mm/s, respectively, compared with those of the base oil. Similarly, in the steady-state, the friction reducing effect of ZrS_2_ is not obvious when compared with the effect during the running-in period. 

In the running-in period, the friction coefficient of all oil samples increased with the increase in velocity. The running-in time of the base oil and that with the addition of 1.0 wt% ZrS_2_ decreased and increased with the increase in velocity, respectively. In the steady-state, the friction coefficient of all oil samples decreased with the increase in velocity.

All results indicate that the addition of ZrS_2_ can effectively decrease the friction coefficient and running-in time in the case of variable velocity or load, indicating its good friction reducing effect in the running-in period.

## 4. Conclusions

In this study, the tribological properties of lamellar ZrS_2_ nanobelts as lubricant additives were investigated. The conclusions are as follows.

The 1.0 wt% concentration of ZrS_2_ yields the best antifriction and antiwear performance. With the addition of 1.0 wt% ZrS_2_, the coefficient of friction decreases by approximately 60% during the running-in period, the running-in time decreases by approximately 67% and the wear volume decreases by 85% when compared with the base oil.

ZrS_2_ can also significantly increase the load-carrying capacity of oil. The 1.0 wt% concentration is the best concentration to improve the load-carrying capacity of oil, represented by PB values that increase by 92% when compared with the base oil.

The addition of ZrS_2_ can effectively decrease the friction coefficient and running-in time in the case of variable velocity or load.

The ZrS_2_ additives can increase the critical temperature of oil from 100 °C to 200 °C, indicating that the ZrS_2_ additives can increase the working temperature of the oil.

## Figures and Tables

**Table 1:** Parameters of the pin-on-disk and ball-on-disk configurations.

	Material	Tensile Strength (MPa)	Yield Strength (MPa)	Hardness	Size (mm)
Ball disk	9Cr18	741	295	58 HRC (Rockwell C Hardness)	*ϕ*10
	1Cr18Ni9Ti	680	265	**158 HB** (Brinell Hardness)	*ϕ*40 × 10

**Table 2:** Material chemical composition of the ball-on-disk configurations (weight%).

	C	Si	Mn	Cr	Ni	S	P
9Cr18	1.03	0.39	0.47	18.3	0.16	0.003	0.008
1Cr18Ni9Ti	0.04	0.12	1.22	17.65	8.24	0.01	0.009
